# Terrestrial mesopredators did not increase after top-predator removal in a large-scale experimental test of mesopredator release theory

**DOI:** 10.1038/s41598-021-97634-4

**Published:** 2021-09-14

**Authors:** Geoff Castle, Deane Smith, Lee R. Allen, Benjamin L. Allen

**Affiliations:** 1 Institute for Life Sciences and the Environment, University of Southern Queensland, Toowoomba, QLD 4350 Australia; 2 NSW Department of Primary Industries, Vertebrate Pest Research Unit, Armidale, NSW 2351 Australia; 3 Department of Agriculture and Fisheries, Queensland Government, Toowoomba, QLD 4350 Australia; 4 Centre for African Conservation Ecology, Nelson Mandela University, Port Elizabeth, 6034 South Africa

**Keywords:** Biodiversity, Conservation biology, Invasive species

## Abstract

Removal or loss of top-predators has been predicted to cause cascading negative effects for ecosystems, including mesopredator release. However, reliable evidence for these processes in terrestrial systems has been mixed and equivocal due, in large part, to the systemic and continued use of low-inference study designs to investigate this issue. Even previous large-scale manipulative experiments of strong inferential value have been limited by experimental design features (i.e. failure to prevent migration between treatments) that constrain possible inferences about the presence or absence of mesopredator release effects. Here, we build on these previous strong-inference experiments and report the outcomes of additional large-scale manipulative experiments to eradicate Australian dingoes from two fenced areas where dingo migration was restricted and where theory would predict an increase in extant European red foxes, feral cats and goannas. We demonstrate the removal and suppression of dingoes to undetectable levels over 4–5 years with no corresponding increases in mesopredator relative abundances, which remained low and stable throughout the experiment at both sites. We further demonstrate widespread absence of negative relationships between predators, indicating that the mechanism underpinning predicted mesopredator releases was not present. Our results are consistent with all previous large-scale manipulative experiments and long-term mensurative studies which collectively demonstrate that (1) dingoes do not suppress red foxes, feral cats or goannas at the population level, (2) repeated, temporary suppression of dingoes in open systems does not create mesopredator release effects, and (3) removal and sustained suppression of dingoes to undetectable levels in closed systems does not create mesopredator release effects either. Our experiments add to similar reports from North America, Asia, Europe and southern Africa which indicate that not only is there a widespread absence of reliable evidence for these processes, but there is also a large and continually growing body of experimental evidence of absence for these processes in many terrestrial systems. We conclude that although sympatric predators may interact negatively with each other on smaller spatiotemporal scales, that these negative interactions do not always scale-up to the population level, nor are they always strong enough to create mesopredator suppression or release effects.

## Introduction

Global biodiversity loss is accelerating and the rate of species extinctions now exceeds the background rate by 2–3 orders of magnitude^1^. Up to 80% of the world’s largest terrestrial predator populations are in decline as a result of habitat loss and fragmentation, human conflict (especially livestock-related conflict), and reductions in prey populations^2^. Because many predators fulfil important ecological functions, such as regulation of herbivory^3,4^, the loss or removal of top-predator populations can be particularly concerning for some ecosystems^5,6^. One major concern is the potential for subsequent increases in the abundance and impacts of mesopredators that are reported to have devastating consumptive and non-consumptive effects on multiple species^7–9^, especially endangered prey species^10^. Mesopredators are expected to be suppressed by larger-sized top-predators, so losing or removing top-predators is expected to produce a ‘mesopredator release’ with undesirable cascading effects on prey fauna at lower trophic levels. Understanding the ecological outcomes of top-predator removal is important for improving predation management practices and stemming biodiversity declines^11,12^.

Dingoes (*Canis familiaris*; a canid) are Australia’s largest terrestrial predator, and one of the two top-predator species in the world (the other is grey wolves, *Canis lupus*) whose ecological roles have been most thoroughly explored^2^. At an average adult body weight of 15.7 kg^13^, dingoes are believed to suppress extant populations of European red foxes (*Vulpes vulpes*; ~ 7 kg; another canid) and feral cats (*Felis catus*; ~ 3 kg; a small felid) (e.g.^8,11,14–17^. All three sympatric species co-occur across most of the continent and have done so since foxes were introduced in ~ 1878 and cats between 1824 and 1886^18^. Dingoes did not evolve in Australia and arrived less than 3,500 years ago^19^, but are nevertheless considered a naturalised native species^20,21^. All three species are generalist predators with overlapping dietary niches which include livestock and threatened native fauna. All three predators are also subject to broad-scale lethal control programs in many places to reduce these impacts^22,23^—control programs which simultaneously target all three predators. However, some have raised concern that controlling or reducing dingoes in this context could have the net effect of releasing or increasing foxes and/or cats that will then go on to exacerbate declines in native fauna populations (e.g.^24–28^). Goannas (varanidae) are similar-sized, native, reptilian mesopredators with generalist diets that might also be released following mammal predator removal^29,30^.

Dingoes are supposed to supress mesopredator abundance through two mechanisms. First, being larger and more dominant in agonistic interactions, dingoes are expected to directly kill mesopredators. Evidence for dingoes killing foxes, cats, and goannas has been largely inferred from the presence of their remains in dingo scat and stomach samples^31–34^, but has also been directly observed in some cases^35^. Second, their broadly overlapping distributions and diet suggests that dingoes may suppress mesopredators through indirect competition for shared prey resources^10,11,33^, though others have pointed out that this simply means all three predators eat the same things and threaten the same species^36^. While these two types of negative interactions undoubtedly occur at fine spatial and temporal scales, reliable evidence for population-level effects at larger spatial and temporal scales has been mixed and equivocal^37,38^. In areas of eastern and northern Australia where bottom-up factors like rainfall and climate are more stable, long-term studies have not found negative relationships between populations of dingoes and mesopredators^39–41^. Thus, negative relationships between dingoes and mesopredators are expected to be strongest and most apparent in arid and semi-arid areas of Australia where the climate is unpredictable and competition for unreliable prey resources is strongest^10^. Frequent drought conditions produce frequent prey shortages which should enhance the suppressive effects of dingoes on mesopredators in these areas^42,43^.

Many studies have investigated these processes (at least 22 literature reviews are listed in^44^). However, after approximately five decades of dingo research there is still no reliable evidence for dingo control-induced mesopredator release due, in large part, to the systemic and continued use of low-inference study designs to investigate the subject^12,37,45,46^. This conclusion is debated by some authors^47–51^. However, what is *not* debated is the fact that almost all the evidence ‘for’ the occurrence of dingo control-induced mesopredator release comes from snap-shot, single-survey or correlative studies, whereas almost all the evidence ‘against’ it comes from large-scale and long-term manipulative experiments of greater inferential value^37,45,52^. Correlative studies are certainly useful for formulating hypotheses about potential causal processes, but they have no power whatsoever for demonstrating causal processes—this is indisputable^53–62^. Thus, not only is there a demonstrable absence of reliable evidence for dingo control-induced mesopredator release, there is also demonstrable evidence of absence for it as well.

For example, Allen and colleagues^63,64^ (but see also^65,66^) experimentally demonstrated that contemporary dingo removal practices (i.e. repeated, temporary reductions in dingo abundance) at multiple sites across Australia did not produce mesopredator releases of foxes, cats or goannas, and cessation of dingo removal practices (i.e. passive increases in dingo abundance) did not produce decreases in fox, cat or goanna abundances either. Theirs was an applied study in open systems to investigate whether or not contemporary predation management practices produced the mesopredator releases feared by some people. They demonstrated that mesopredator releases did not occur because dingo populations quickly recovered following each removal event, so the ‘trophic cascade’ never got a chance to begin. Johnson^50^ (see also response by^67^) quite rightly observed that the unmanaged immigration or post-control recovery of dingo populations in those experiments meant that they were not a strict test of mesopredator release theory, recommending that future inferences about the absence of mesopredator release could be improved by experimentally controlling for dingo migration. Newsome et al.^68^ reinforced this view, affirming that uncertainty about dingo effects on mesopredators could be resolved if experiments were conducted in closed or fenced ecosystems where dingo numbers can be sufficiently and sustainably reduced and the ecological outcomes observed. Allen and colleagues’ experiments^63,64^ were the second-largest predator manipulation experiments in the world, and the largest and strongest-inference experiments ever conducted on the subject in Australia. But addressing the migration issues raised by Johnson^50^ and Newsome et al.^68^ required fortifying the levels of inference even further by conducting similar experiments in closed, fenced systems where dingo populations can be manipulated more effectively.

Implementation of such predator manipulation experiments had already begun, and are described here for two fenced sites (Morven and Tambo) used for extensive livestock production in a semi-arid area of Australia. Our goal was to eradicate dingo populations inside the two fenced areas and give foxes, cats and goannas a chance of being freed from any suppressive effects that dingoes might impose on them. At each site we compared the fenced treatment areas to adjacent, paired nil-treatment areas outside the fences, and simultaneously monitored populations of dingoes, foxes, cats and goannas over 4–5 successive years. We repeatedly sampled predator populations through spotlighting (density estimates) and passive tracking indices (PTI; relative abundance estimates). In this context, mesopredator release theory predicts that foxes, cats and/or goannas would increase inside the fences—relative to outside the fences—in response to the demonstrated removal of dingoes inside the fences. We therefore predicted that:

1. Dingo PTI would be, on average, lower inside the fences than outside;
2. Mesopredator PTI would be, on average, higher inside the fences than outside;
3. Dingo control would supress or remove dingoes inside the fenced areas, but not outside;
4. Mesopredator PTI trends would increase inside the fenced areas relative to the paired outside nil-treatment areas over time; and
5. Negative relationships would be apparent between dingoes and mesopredators.

Our experiments and analyses closely follow the ‘classical experiment’ approach most recently applied by Allen et al.^63,64^ and recommeded by Glen et al.^69^, Johnson et al.^50^ and Newsome et al.^68^; although in the present case, our treatment areas were also fenced with dingo-proof fencing to prevent migration of dingoes back into controlled areas post-control, thereby facilitating their sustained removal inside the fences. We assess overall mean PTI differences between treatments (inside vs. outside); evaluate the success of dingo removal efforts inside the fences (i.e. demonstrate a treatment effect); assess the responses of mesopredator PTI to dingo removal over time, after accounting for normal seasonal variation in predator activity (i.e. determine if mesopredator releases occurred); and evaluate the relationships between dingoes and mesopredators (i.e. verify the presence of the proposed mechanism). Finally, we summarise the status of manipulative experimental research on dingo-control induced mesopredator release and offer some guidance for future studies investigating the ecological outcomes of top-predator removal or introduction.

## Results

Spotlighting efforts produced insufficient data on predator populations for any meaningful analyses (Table 1; see also^70^), so we focussed all our analyses on data obtained from passive tracking indices.

**Table 1:** Total number of dingoes, foxes, feral cats and goannas observed during the entire study period.

	Plotnights	Dingo		Fox		Cat		Goanna	
		Sandplot	Spotlight	Sandplot	Spotlight	Sandplot	Spotlight	Sandplot	Spotlight
**Morven**									
Inside	2,358	64	5	17	0	39	42	11	0
Outside	2,180	282	2	0	1	21	14	23	0
**Tambo**									
Inside	2,128	161	0	1	3	35	41	3	0
Outside	1,818	208	3	1	0	11	29	6	0

Sandplots represent the number of individual animal tracks or footprints observed. Spotlighting represents the number of individual animals observed.

### Overall patterns in relative abundance

**Figure 1:**
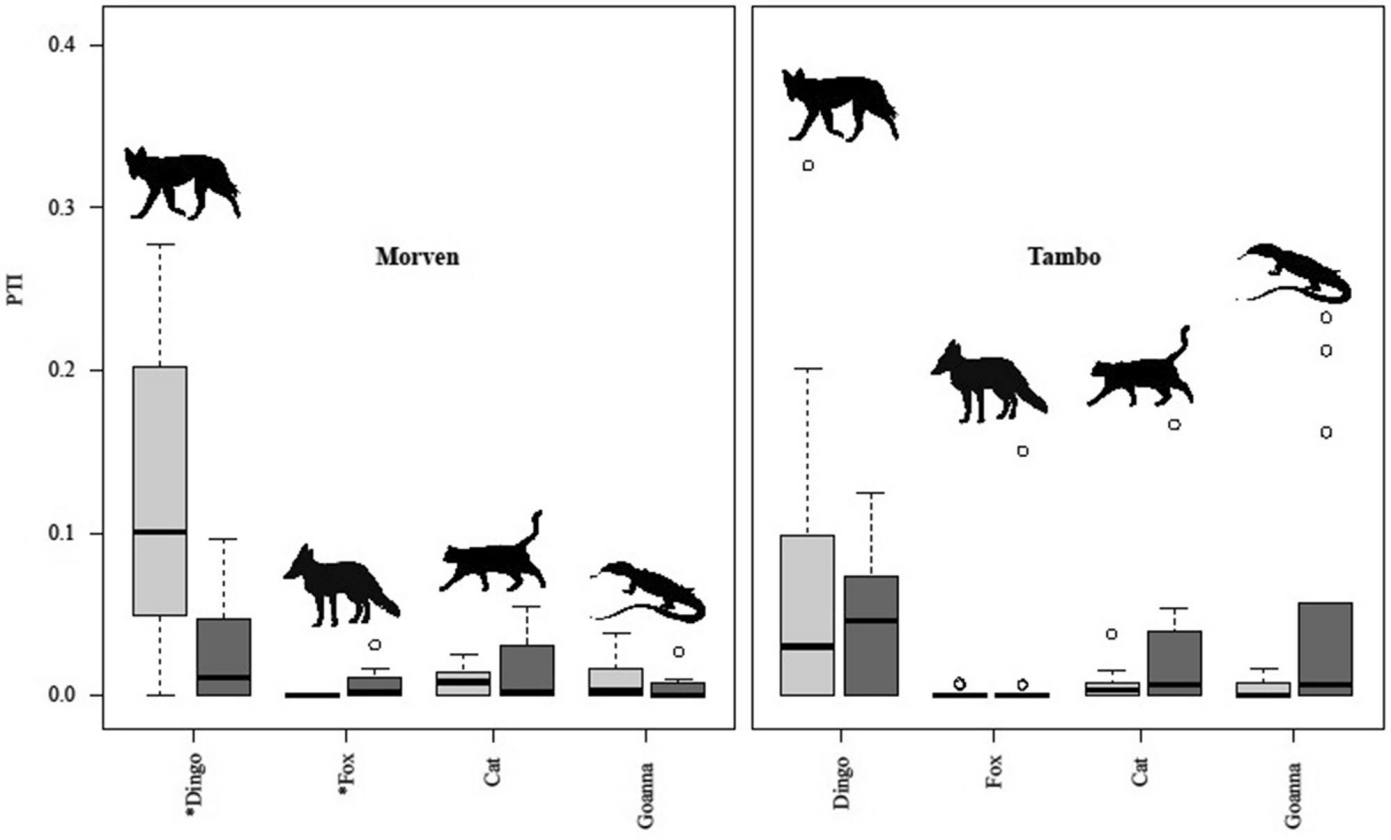
Overall mean PTI values for dingoes and sympatric mesopredators at Morven and Tambo. PTI values are from all surveys outside (light grey) and inside (dark grey) fenced areas. *Denotes demonstrable differences, see Table 2 for details.

Dingoes, foxes, cats and goannas were present both inside and outside the fences at both sites; each were detected on sand plots in both treatments at both sites, although foxes outside the fence at Morven were only detected during spotlighting (Table 1). Welch’s T-tests indicated that overall mean dingo PTI was lower inside the fence at Morven (t = −3.579, df = 17, p = 0.002) where fox PTI was higher (t = 2.721, df = 13, p = 0.018). Cat PTI appeared higher inside the fence at Tambo (t = 1.997, df 22, p = 0.059). We found no other differences in overall mean PTI for any predator species at either site (Table 2, Fig. 1). Thus, the greater overall relative abundance of foxes inside the fence at Morven and cats inside the fence at Tambo were the only instances (of six possible site x mesopredator combinations) where a sympatric mesopredator was detected more frequently inside the fence at either site.

**Table 2:** Overall mean predator PTI values inside and outside fenced areas at Morven and Tambo.

	Predator	Inside PTI mean	Outside PTI mean	t	df	p
Morven	*Dingo*	*0.03*	*0.12*	*− 3.5786*	*17*	*0.0024*
	*Fox*	*0.01*	*0.00*	*2.7205*	*13*	*0.0175*
	Cat	0.02	0.01	0.9577	18	0.3510
	Goanna	0.00	0.01	*− *1.2185	22	0.2362
Tambo	Dingo	0.08	0.12	*− *1.3182	19	0.2028
	Fox	0.00	0.00	*− *0.1061	25	0.9163
	*Cat*	*0.02*	*0.01*	*1.9972*	*22*	*0.0585*
	Goanna	0.00	0.00	*− *1.0996	18	0.2857

Results of Welch’s two-tailed t-tests for differences in overall mean predator PTI between treatments (see also Fig. 1). Demonstrable differences in *italics.*

### Evidence of a treatment effect

We could not quantify all forms of dingo control undertaken at each site (i.e. repeated poison baiting, trapping, and shooting) to confirm exactly how many predators were removed and precisely when they were removed, but we were able to ascertain some accurate information on dingo control effort.

**Figure 2:**
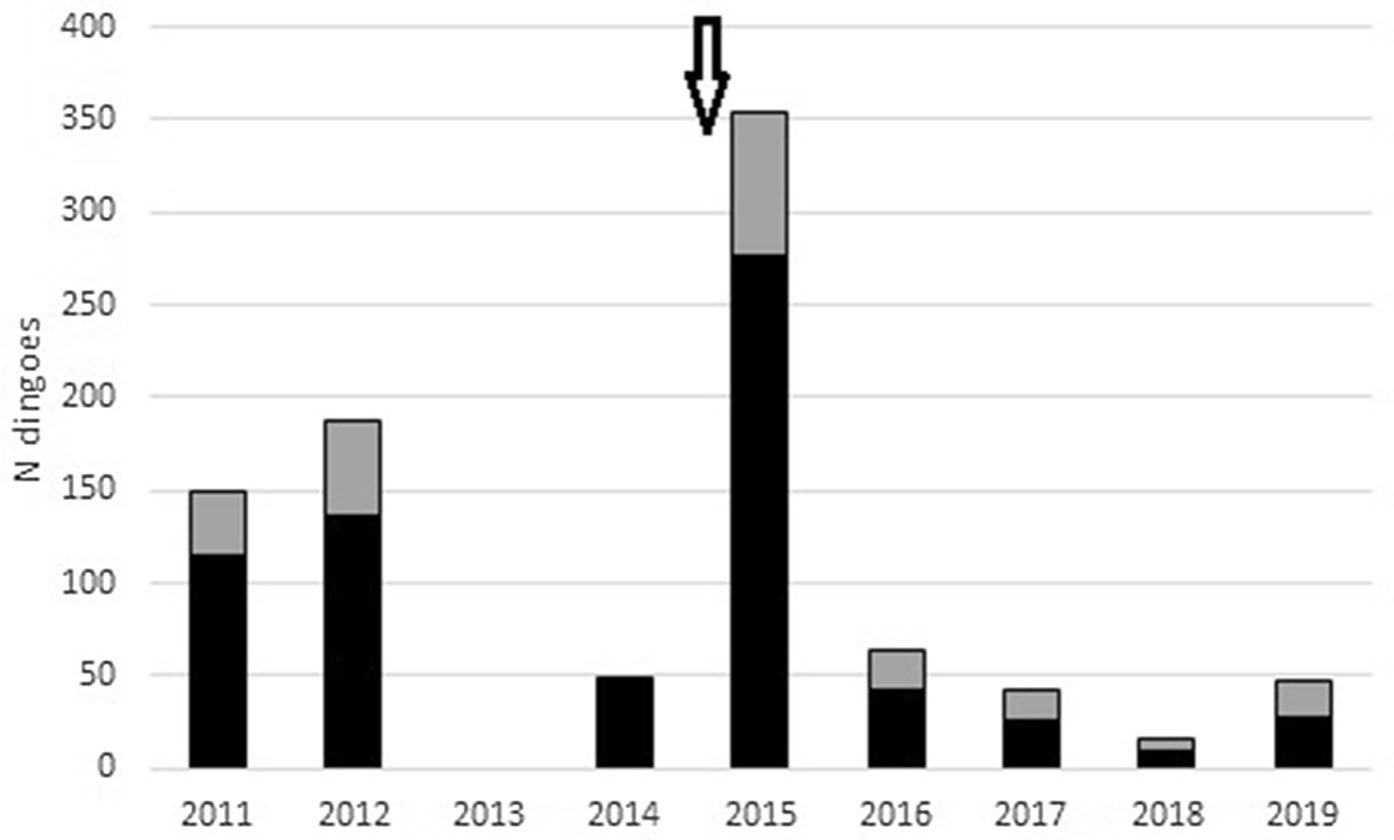
Dingo removal effort at Morven. The number of landholder-reported adult (black) and pups/juvenile (grey) dingoes trapped and removed from inside the fenced treatment area at Morven, 2011 to 2019. Dingo control effort varied over time, but was most intensive and fairly consistent between 2015 and 2019. The arrow denotes the approximate date when the fence was completely closed.

Livestock producers at Morven reported that all livestock properties engaged dingo trappers on at least one occasion each year, and many of them engaged trappers on multiple occasions each year. Self-reported trapping records from Morven indicated that at least 906 dingoes were trapped and removed from inside the fenced area between 2011 and 2019, of which 226 (25%) were pups or juveniles (Fig. 2). However, the dingo exclusion fence was still under construction in the early part of this period, and the fence was not completely sealed until January 2015. A rapid and substantial increase in control effort followed the completion of the fence, and livestock producers reported the subsequent removal of 354 dingoes in the first year after fence completion. In addition to this trapping effort, all properties inside the fence at Morven distributed poisoned baits twice each year, except for one property which baited five times each year. Like baiting, trapping at Tambo was less intensive, and yielded only 250 dingoes between 2016 and 2019. Trappers did not report trapping or shooting any foxes, cats or goannas during the study at either site.

**Figure 3:**
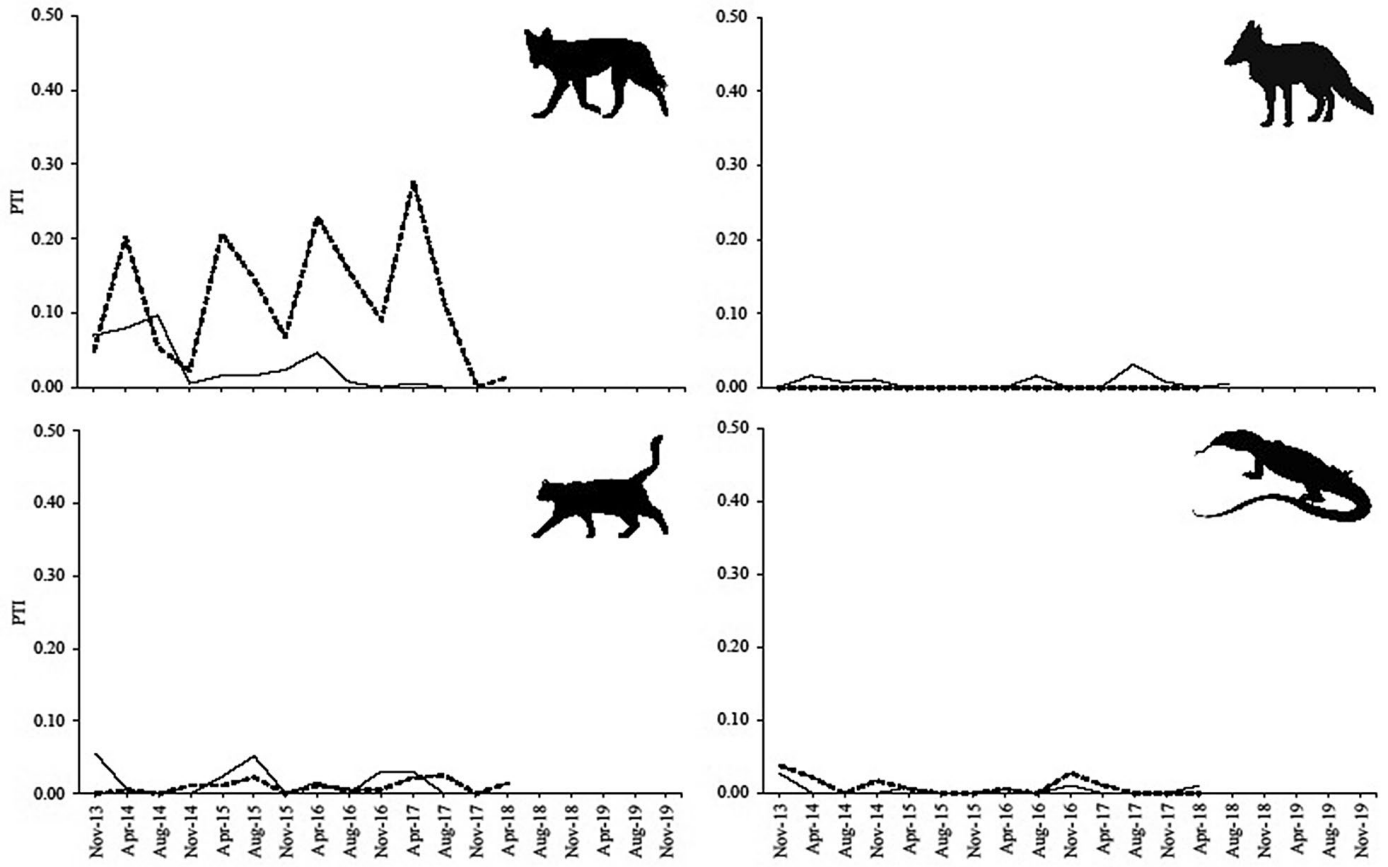
PTI trends of dingoes, foxes, cats, and goannas at Morven, 2013–2018.

**Figure 4:**
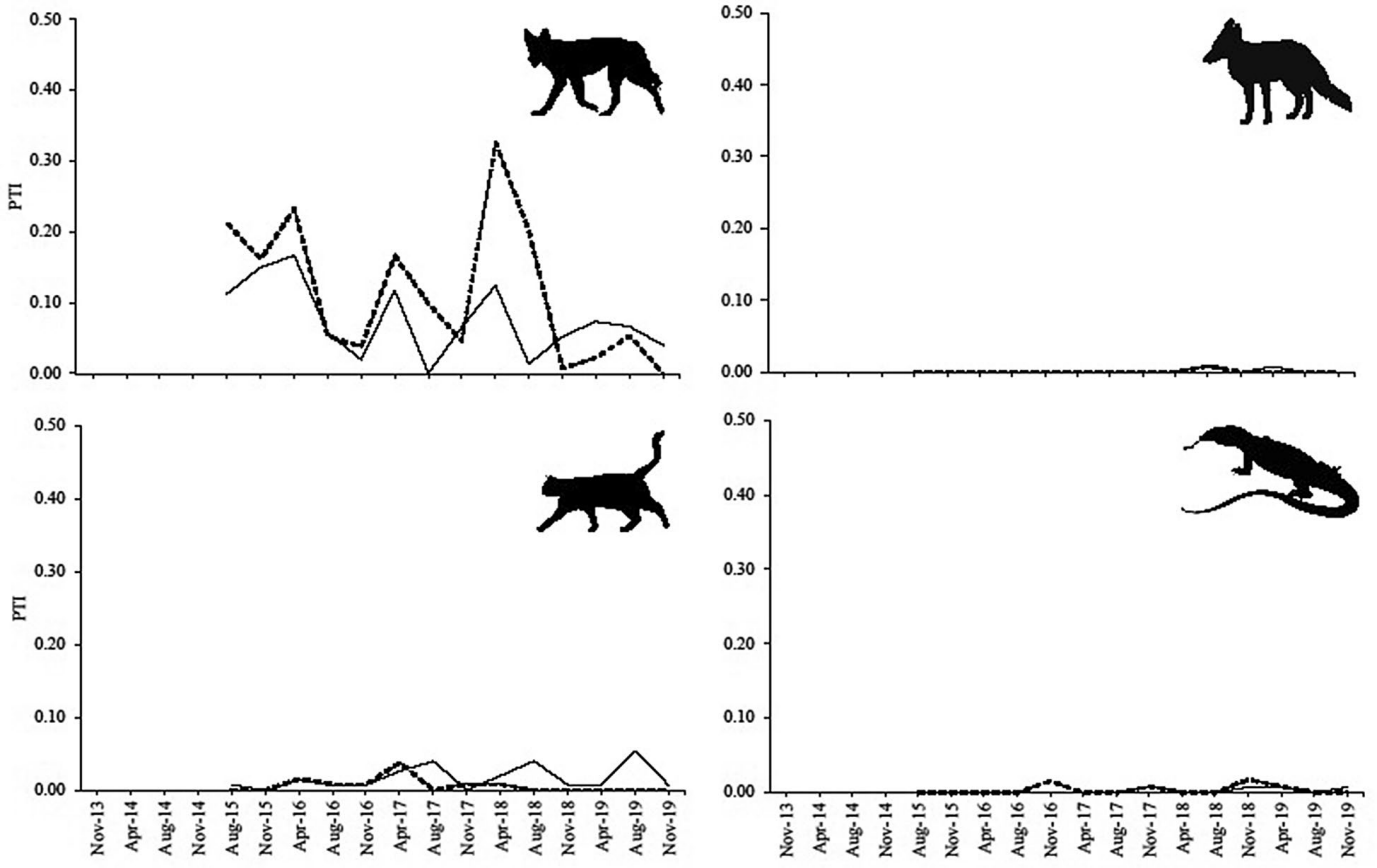
PTI trends of dingoes, foxes, cats, and goannas at Tambo, 2015–2019.

**Figure 5:**
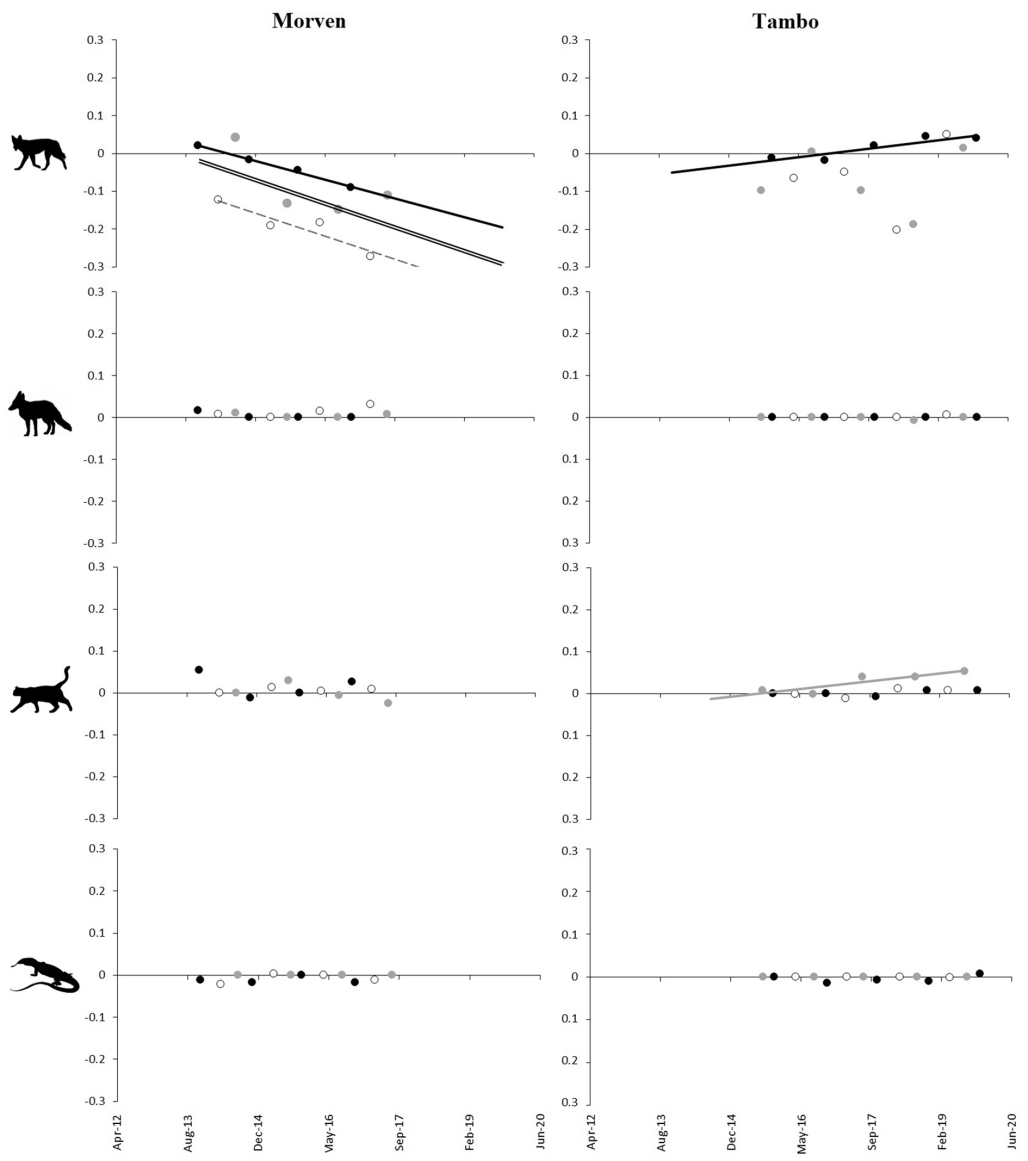
Temporal trends in dingo, fox, cat and goanna PTI treatment differences at Morven and Tambo. Black marks and black lines = November (spring) surveys, hollow marks and dashed line = April (autumn) surveys, and grey marks and grey line = August (winter) surveys. Black double-line = all seasons combined. Ascending slopes denote increases inside the fence relative to outside, whereas descending slopes denote decreases inside the fence relative to outside. See Table 3 for further details.

These dingo control efforts are reflected in the PTI trends which suggest a marked decline of dingoes inside the Morven fence between 2015 and 2016, with sustained suppression of dingoes at undetectable levels thereafter (Fig. 3). Outside the fence at Morven, dingo PTI increased over time while fluctuating with seasonal peaks in autumn (mating season) and troughs in spring (whelping season)—a predictable and normal seasonal activity pattern widely expressed by dingo populations across the continent^21,22^. Dingo PTI at Tambo likewise fluctuated seasonally both inside and outside the fence (Fig. 4), although dingo control efforts at this site were apparently not as effective at supressing dingoes (Fig. 4). At Tambo, dingo PTI steadily declined over time both inside and outside the fence. After accounting for seasonal influences on our data and assessing differences in dingo PTI trends between inside or fenced and outside or unfenced areas (Table 3, Fig. 5), we found that dingoes were indeed reduced and sustainably supressed inside our treatment area at Morven (autumn data R^2^ = 0.86, p = 0.072; spring data R^2^ = 0.99, p = 0.004; combined data R^2^ = 0.36, p = 0.040). This did not occur at Tambo, where dingoes declined slower inside the fence then they did on the outside (spring data R^2^ = 0.82, p = 0.034).

**Table 3:** Temporal trends in dingo, fox, cat and goanna PTI treatment differences at Morven and Tambo.

Site	Predator	Season	R-squared	F and P values
Morven	Dingo	*Autumn*	*0.86*	*F(1,2) = 12.329, p = 0.072*
		*Spring*	*0.99*	*F(1,2) = 273.741, p = 0.004*
		Winter	0.50	F(1,2) = 2.015, p = 0.291
		*Combined*	*0.36*	*F(1,10) = 5.579, p = 0.034*
	Fox	Autumn	0.70	F(1,2) = 4.757, p = 0.161
		Spring	0.60	F(1,2) = 2.969, p = 0.227
		Winter	0.05	F(1,2) = 0.101, p = 0.781
		Combined	0.02	F(1,10) = 0.164, p = 0.694
	Cat	Autumn	0.19	F(1,2) = 0.472, p = 0.563
		Spring	0.11	F(1,2) = 0.241, p = 0.672
		Winter	0.39	F(1,2) = 1.289, p = 0.374
		Combined	0.15	F(1,10) = 1.812, p = 0.208
	Goanna	Autumn	0.13	F(1,2) = 0.293, p = 0.642
		Spring	0.00	F(1,2) = 0.005, p = 0.948
		Winter	1.00	F(1,2) = NA, p = NA
		Combined	0.07	F(1,10) = 0.738, p = 0.41
Tambo	Dingo	Autumn	0.0563	F(1,2) = 0.119, p = 0.763
		*Spring*	*0.8226*	*F(1,3) = 13.907, p = 0.034*
		Winter	0.0034	F(1,3) = 0.01, p = 0.925
		Combined	0.0536	F(1,12) = 0.679, p = 0.426
	Fox	Autumn	0.5943	F(1,2) = 2.93, p = 0.229
		Spring	1.0000	F(1,3) = NA, p = NA
		Winter	0.1294	F(1,3) = 0.446, p = 0.552
		Combined	0.0042	F(1,12) = 0.051, p = 0.825
	Cat	Autumn	0.3959	F(1,2) = 1.311, p = 0.371
		Spring	0.3070	F(1,3) = 1.329, p = 0.332
		*Winter*	*0.8125*	*F(1,3) = 12.998, p = 0.036*
		Combined	0.2112	F(1,12) = 3.213, p = 0.098
	Goanna	Autumn	0.5943	F(1,2) = 2.93, p = 0.229
		Spring	0.1049	F(1,3) = 0.351, p = 0.595
		Winter	1.0000	F(1,3) = NA, p = NA
		Combined	0.0185	F(1,12) = 0.226, p = 0.643

Demonstrable differences in *italics*. See Fig. 5 for further details.

### Evidence of mesopredator release

We found no evidence of fox, cat, or goanna PTI increases inside fenced areas where dingoes had declined (Figs. 3 and 4)—a result reinforced by the almost complete absence of any PTI divergence between treatments for any mesopredator (Fig. 5). Fox, cat and goanna PTI trends were essentially identical inside and outside dingo-fenced areas at both sites (Figs. 3, 4, 5). Each of these mesopredator species were present inside the fence at the beginning of the study at both Morven and Tambo, but by the end of the study 4–5 years later each had failed to increase inside the fence (relative to outside) despite dingo control efforts demonstrably eliminating and supressing dingo populations, at least at Morven. The only possible exception to this was cats at Tambo. At this site, analyses of data from the winter surveys suggested that cat activity peaks in winter increased over time, but this trend was not detectable when using the autumn or spring data, or all the data combined (Table 3, Fig. 5).

### Relationships between dingoes and mesopredators

**Figure 6:**
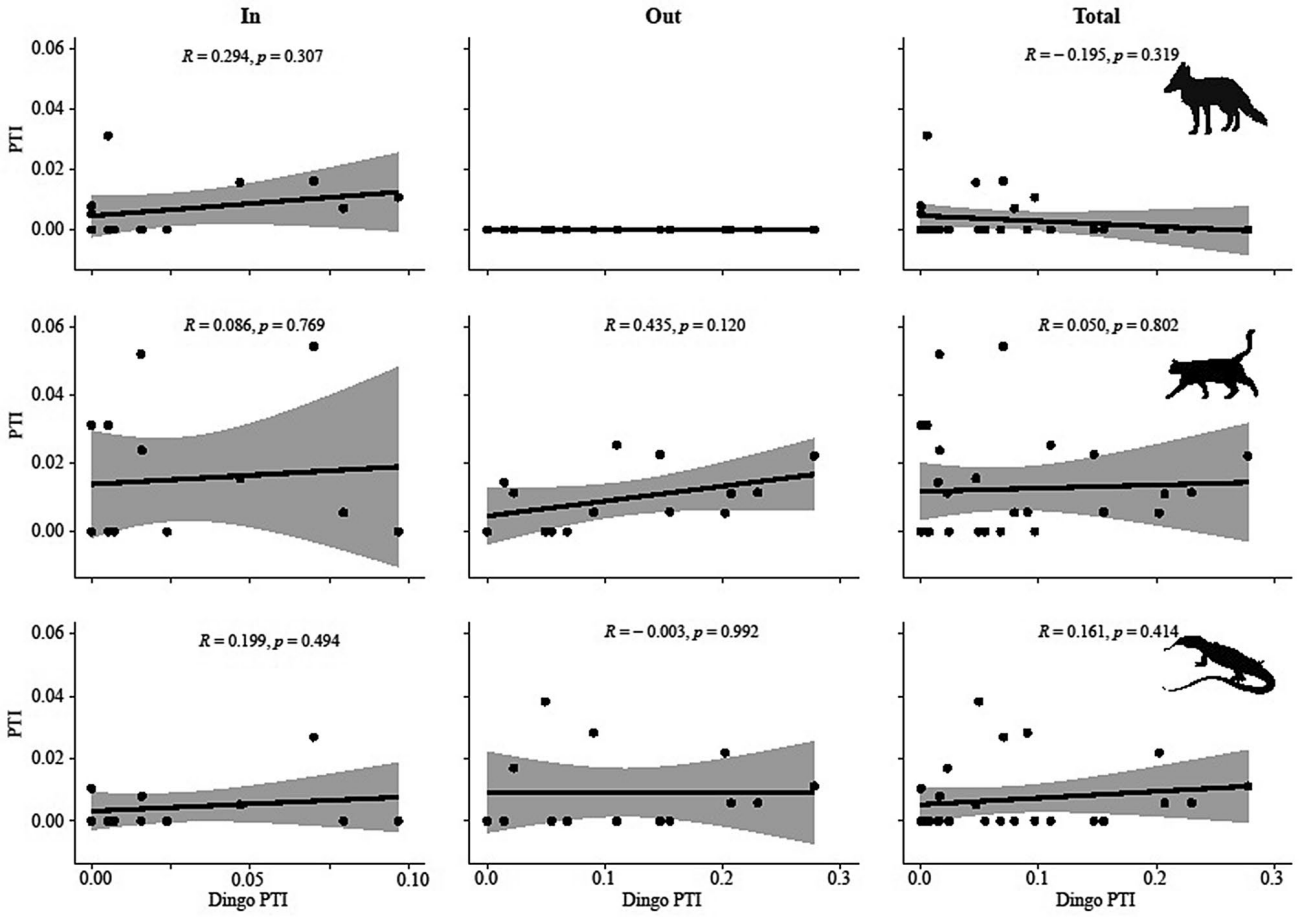
Relationships between dingo PTI and fox, cat and goanna PTI at Morven. Shading denotes 95% confidence intervals. Pairwise correlations (r) and p values also shown. Note that the scales are inconsistent between panels and have been adjusted to allow closer inspection of the data.

**Figure 7:**
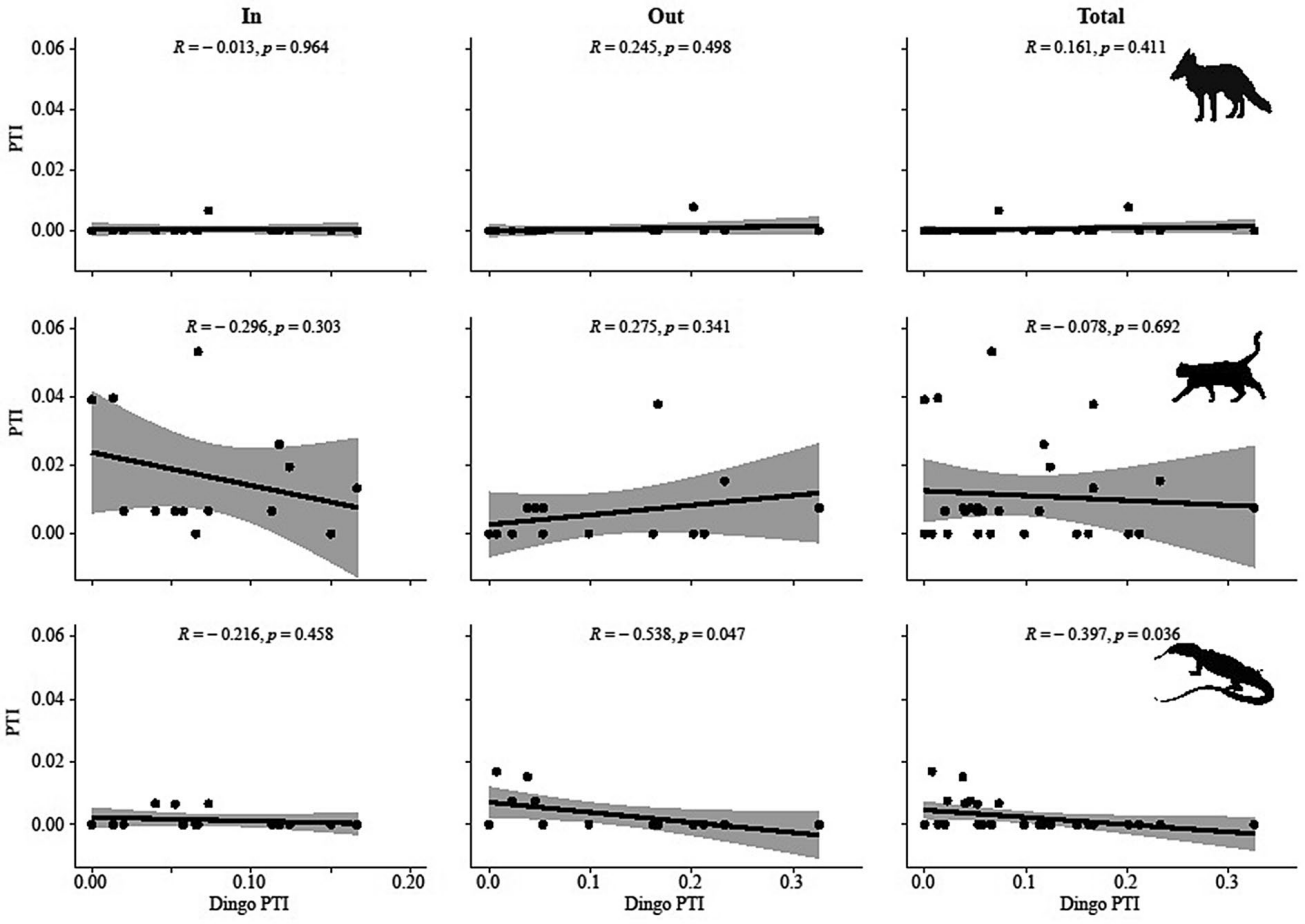
Relationships between dingo PTI and fox, cat and goanna PTI at Tambo. Shading denotes 95% confidence intervals. Pairwise correlations (r) and p values also shown. Note that the scales are inconsistent between panels and have been adjusted to allow closer inspection of the data.

We found no relationships—negative or positive—between dingoes and either foxes or cats at Morven, whether inside the fence, outside the fence, or pooling the data and ignoring the fence altogether (Fig. 6). We likewise found no relationships—negative or positive—between dingoes and either foxes or cats at Tambo, whether inside the fence, outside the fence, or pooling the data and ignoring the fence altogether (Fig. 7). We did detect a positive relationship between cats and goannas inside the fence at Morven (r = 0.566, p = 0.035) and a positive relationship between foxes and goannas inside the fence at Tambo (r = 0.536, p = 0.048). But the only negative relationship we detected was between dingoes and goannas outside the fence at Tambo (r = −0.538, p = 0.047), which was also detectable when data was pooled and fences were ignored (r = −0.397, p = 0.036). All other pairwise relationships between dingoes, foxes, cats and goannas indicated that predator relative abundances fluctuated independently of each other (Figs. 6 and 7). Fox, cat and goanna PTI remained relatively low and stable regardless of whether or not dingo PTI was low or high. In other words, mesopredator population trends fluctuated independently of dingo population trends over time both inside and outside the fences.

## Discussion

Mesopredator release theory predicts that the removal or loss of top-predators will cause numerical increases in mesopredators^7,8^, but we found no evidence for these processes in our experiments. Neither goannas, cats, nor foxes increased inside the fence at Morven despite the sustained removal and suppression of dingoes there (Figs. 3 and 5). Initial lethal control efforts (Fig. 2) demonstrably depopulated the area inside the fence of dingoes (Figs. 3 and 5) and ongoing control efforts held them at near-undetectable levels over subsequent years while their relative abundance increased outside the fence (Fig. 3). Yet despite the sustained removal of dingoes throughout the fenced area, the extant mesopredator populations inside the fenced area at the beginning of the study failed to increase over time. Similarly, neither goannas nor foxes increased inside the fenced area at Tambo despite substantial declines (about a 60% reduction) of dingoes inside the fence at that site (Fig. 4). However, we could not attribute this decline solely to dingo control efforts because dingo PTI trends declined slower inside the fence than they did on the outside, implying a relative increase of dingoes inside the fence at Tambo (Fig. 5). Cats also failed to increase inside the fence at Tambo, although there was some suggestion that their winter-time activity peaks increased over time (Table 3, Fig. 5). Regardless, this possible increase in cat activity cannot be considered evidence of dingo control-induced mesopredator release given that dingoes at Tambo also increased inside the fence relative to outside (Fig. 5; i.e. a positive relationship between dingoes and cats). Results from both sites therefore provide no evidence of mesopredator release following dingo control and removal.

### Overall PTI differences between treatments

After pooling data from all 14 surveys together to assess overall treatment differences in mean PTI, we found few differences for any predator except for foxes and dingoes at Morven, and cats at Tambo (Table 2, Fig. 1). At Morven, dingoes were lower inside the fence and foxes were higher (Fig. 1, Table 2). We expected overall mean dingo PTI to be lower given the intensive dingo control efforts inside the fence there. Dingo PTI at Morven ranged between 0.07 and 0.10 (i.e. one dingo track observed every 7–10 sand plots, on average) in the first year of the study, but was zero (not a single dingo track observed) throughout the entire last year of the study (Fig. 3). Though the difference in overall mean fox PTI might at first be considered a tantalising suggestion of a possible mesopredator release, this was instead an artefact of our sampling given foxes were not recorded on sand plots outside the fence on any occasion at Morven (Table 2). In other words, the Welch’s two-tailed T-test was not functioning as a test for differences between PTI means in this case, but rather as a one-tailed T-test for a difference between a mean and zero. This test result essentially indicates that there were more than zero foxes inside the fence at Morven, which we had already known at the beginning of the study (Fig. 3) and is not particularly noteworthy. Moreover, and despite being a common way of searching for and claiming evidence of mesopredator releases (for examples, see^24,26,71–73^), such simplistic and correlative comparisons of overall mean PTI between treated and untreated areas cannot and do not elucidate any causal processes and hence cannot be used to make inferences about dingo control-induced mesopredator release^45^ (but see also^53–62^).

The lower mean PTI of dingoes inside the fence at Morven (Fig. 1) coupled with demonstration of a treatment effect (Table 3, Fig. 5) confirms our Prediction 1 that the relative abundance of dingoes would be lower in places with intensive and sustained dingo control effort. However, the almost complete absence of higher mesopredator mean PTI values inside the fenced areas at each site did not accord with our Prediction 2 that mesopredator abundances would be higher in places where top-predators are reduced or absent.

### Evidence of a treatment effect, dingo control effectiveness

There are a few possible ways of gauging the effectiveness of dingo control from the data in our study, or confirming a treatment effect. For this study we define effectiveness as the complete eradication or near-eradication of dingoes, or some sort of demonstrable reduction in dingo PTI. Like others we might consider an overall mean dingo PTI difference between treatments as evidence of a treatment effect (cited above), but alone, that would be a very weak approach. A far stronger approach is to assess divergence of PTI trends over time after accounting for normal seasonal influences on PTI variation^67,74^. Using only differences in mean PTI between treatments, we might conclude that dingo control was effective at Morven but not at Tambo (Fig. 1, Table 2). But closer inspection of Fig. 3 concurs and indicates that dingoes were declining inside the fence at the same time they were increasing outside the fence at Morven. Statistical support for this result was apparent using autumn data, spring data, and all data combined (Table 3, Fig. 5). Hence, the hundreds of dingoes removed from inside the fence at Morven by intensive and sustained baiting, trapping and shooting (Fig. 2) did indeed cause a demonstrable reduction in dingoes there, confirming a treatment effect. This does not often occur outside fences^75,76^, but can occur in such open systems when control is particularly intensive^22,77^.

Dingo control effectiveness at Tambo was not as pronounced given the lower spatial and temporal intensity of dingo control effort there, including the presence of livestock producers inside the fence that did not undertake any dingo control. Although dingo PTI at Tambo declined by 41–73% inside the fence over the study period (depending on which season was used to measure the decline; Fig. 3), this decline occurred more slowly than it did outside the fence (Fig. 5). This implies a net increase or ‘release’ of dingoes inside relative to outside, and means that no treatment effect could be confirmed at Tambo. We suspect that the dingo control efforts occurring on both sides of the fence at Tambo were sufficient to reduce dingoes to some degree, but that control efforts inside the fence were clearly not intensive enough to generate demonstrable suppression of dingoes beyond the background levels observed outside the fence, as observed in other studies^78^.

These results partially support our Prediction 3, demonstrating that dingo control can effectively reduce dingoes to functionally extinct or undetectable levels inside fences when control efforts are intensive, but not when control efforts are relaxed. Given that Morven was the only site where we demonstrated a treatment effect (Fig. 5), theory would predict that mesopredator releases would be more apparent at Morven than Tambo (discussed below).

The declines and suppression of dingoes at Morven and Tambo also highlight the value of the exclusion fencing erected at our sites (see also^78–81^). Multiple previous studies have demonstrated that even though dingo populations can be temporarily supressed by substantial amounts up to 100% in open or unfenced systems^22,63,77^, they typically recover to pre-control levels within a few months or by the next annual breeding season due to immigration by dispersing dingoes looking for a new home range^82,83^. That dingo populations at Morven were knocked down and then held down inside the fence while dingo populations were increasing outside the fence (Fig. 3) is a powerful demonstration of the utility of cluster fences at excluding dingoes, much like other types of fences exclude other species^79^. This is an important feature of our results because some authors have asserted that even small reductions in dingo abundance produce disproportionate and cascading negative effects on ecosystems through mesopredator release effects. For example, a review of field studies by Ritchie and Johnson^8^ concluded that every unit of decreased top-predator abundance leads to a fourfold increase in mesopredator abundance. The repeated temporary reductions of dingo abundance of 50–70% reported in Allen et al.^63^ were insufficient to generate such predicted mesopredator releases^50^, however, so the threshold level of dingo reductions required are clearly far higher than previously thought (see^10^). Yet Newsome et al.^68^ go further and assert that before dingo control-induced mesopredator releases can be confidently demonstrated not to occur, experiments seeking to empirically test mesopredator release theory must use fenced areas where dingoes might be sustainably supressed to extremely low or undetectable levels. The experimental conditions and results from Morven meet these study conditions and produce the best opportunity yet to experimentally test the theory that contemporary dingo removal produces mesopredator releases.

### Evidence for mesopredator release

We found no evidence of mesopredator releases following demonstrated dingo removal and suppression. Like the results from all previous manipulative experiments in open systems^63–66,84,85^, not only was the overall mean PTI of mesopredators typically no different between treatments at both sites (Table 2, Fig. 1), but mesopredator PTI trends also fluctuated independently of dingo PTI at both sites over time (Figs. 3 and 4). Divergence analyses further failed to yield any evidence of mesopredator release of foxes, cats or goannas (Fig. 5) despite demonstrably reducing dingoes (Table 3) and holding them at undetectable levels at Morven (Fig. 3). The substantial declines of dingoes at Tambo, though not completely attributable to dingo control, likewise failed to generate mesopredator releases (Fig. 5). These results do not accord with our Prediction 4 that mesopredators would increase following a decrease of dingoes.

Foxes, cats and goannas were present inside the fence at both sites at the beginning of the study (Table 1), so the absence of mesopredator releases cannot be attributable to their physical absence. Goannas and cats, and to a lesser extent foxes, are also not impeded by the exclusion fencing used at our sites, so the absence of mesopredator releases cannot be attributable to disruption of mesopredator immigration opportunities potentially caused by the fencing. Dingoes were demonstrably reduced to undetectable levels inside the fence at Morven and evidently could not recover (Fig. 3) either through immigration or compensatory breeding given their ongoing removal (see also^83^), so the absence of any mesopredator releases at that site cannot be attributable to the absence of a treatment effect. Dingo populations inside the fence were reduced and held at undetectable levels while their relative abundance demonstrably increased year-on-year outside the fence, so the absence of mesopredator releases cannot be attributable to an insufficiently long study period needed to observe population-level predator abundance changes, which we were able to observe. The increasing dingo population outside the fence at Morven also suggests that environmental conditions in the region were able to support predator increases, at least until the final year of the study, so the absence of any mesopredator releases cannot be confidently attributable to unsatisfactory environmental conditions either (likewise found by^39,40^). And even if environmental conditions were considered poor, the number of available prey per capita of mesopredators should have increased given the removal of dingoes which share the same prey resources, producing conditions meant to exacerbate mesopredator releases^10,11,33^. Dozens of studies (listed in^38,86^) have also reported road-based passive tracking indices to be a reliable means for detecting mesopredator releases, so the absence of mesopredator releases cannot be attributable to an inability of our survey techniques to detect such changes^51,87,88^. Given that our experiment was executed in a way and at a time and place where mesopredator releases should have been detected if they occurred, we can think of no reason for the consistent absence of any detectable mesopredator releases other than a true absence of any population-level suppressive effects of dingoes on mesopredators.

### Relationships between dingoes and mesopredators

Evidence for inverse relationships between dingoes and mesopredators is absent in most studies (for examples, see^38,41,85,89^), although enough studies (reviewed in^10,11,68,69,90^) and passionate advocacy by authors (for examples, see^14,16,91,92^) have reported inverse relationships to create the romantic religious belief among many people that dingoes supress mesopredators. Unfortunately however, all studies reporting inverse relationships are correlative (akin to our analyses in Table 2 and Fig. 1) and therefore have no power to identify any causal processes, including dingo control^37,45^, so these beliefs are not grounded in strong evidence. Despite a great deal of discussion and excitement on the subject, at the time of writing there is still not a single available study that shows a demonstrable increase of foxes or cats in response to demonstrable decrease in dingoes, or vice versa—studies with both a treatment and an experimental control site where predator numbers have been measured both before and after the treatment or over time, and where the predicted treatment effects and mesopredator responses have been measured and demonstrated. Thus no experimental evidence for dingo control-induced mesopredator releases presently exists in either open systems^63–66,84^ or closed systems (as reported here), but for completeness we nevertheless assessed pairwise relationships between dingoes and mesopredators to search for the presence of the proposed ecological mechanism and help understand why the predicted mesopredators releases are not occurring.

Similar to most other studies we likewise found very little evidence of negative relationships between dingoes and mesopredators at either site (Figs. 6 and 7). Of the four relationships (out of a possible 36 pairwise combinations) we did detect, two were positive, two were negative, and none were between dingoes and foxes or cats. These results were consistent inside fenced areas, outside fenced areas, and after pooling data together from both inside and outside fenced areas at both Morven and Tambo, revealing that our Prediction 5 was also not supported by our data. This absence of any reliable evidence for negative relationships between dingoes and mesopredators^37,38^ may be the underlying reason behind the failure of dingo control to produce any mesopredator releases. Despite observations that dingoes share the same prey resources as mesopredators^31,33,34^ and dingoes occasionally kill mesopredators in agonistic interactions^35^, there is now a large and continually growing body of robust experimental evidence that these interactions do not scale-up to population level effects of dingoes on mesopredators in open or closed systems.

Our experiments focussed on the numerical responses of mesopredators to the removal of dingoes, and we did not directly assess the non-consumptive effects of dingoes on mesopredators. Some propose that a landscape of fear also exists between predators sufficient to drive trophic cascades even in the absence of demonstrable numerical effects^93,94^, or what Haswell et al.^95^ describe as a ‘behaviourally-mediated trophic cascade’. For example, Colman et al.^96^ and Brook et al.^24^ both assert that fox and cat activity should increase even if their populations are not affected numerically. Our data were not intended to measure any psychological fear effects dingoes may have on mesopredators, but even if these fear effects do occur, they did not manifest themselves as mesopredator activity increases within five years after the fencing and subsequent removal of dingoes (Figs. 3, 4, 5; see also^97^). This suggests that dingoes do not create such fear effects at the population-level, or, that dingoes create such profound and lasting fear effects that they can exclude and supress mesopredator activity long after complete dingo removal. We believe this latter conclusion is unsupportable given that foxes and cats colonised Australia in the presence of dingoes^18^ and all three species presently coexist across most of the continent^28^. That dingoes and goannas have coexisted for thousands of years and all the dingoes in Australia could not stop foxes or cats from spreading across the continent following their introduction supports the experimental results of the present study and others that dingoes do not exhibit strong mesopredator-suppressive qualities. Unfortunately, they all appear to coexist rather well, as they do on the continents where they evolved together before they were each brought to Australia^20,98^. We believe that the most parsimonious explanation for these observations and our experimental results is that dingoes do not suppress mesopredators at the population or community level, but additional data beyond our experiments is required to better explore potential non-consumptive effects of dingoes on mesopredators and what these might mean for fauna at lower trophic levels^85,99^.

Based on previous experience we anticipate that some might question the reliability of our experimental results on grounds that: (1) passive tracking indices are unsuitable for monitoring changes in the relative abundance of dingoes, foxes, cats or goannas; that (2) count data like ours must be somehow transformed before it can be properly analysed; that (3) our analytical procedures are uninformative and we should have instead used occupancy modelling, quantile regressions, or some other form of modelling; that (4) we should have setup our analyses of the available experimental treatments and controls in some other way; or that (5) such applied, experimental tests of mesopredator release theory are somehow invalid because dingo control practices are known to also kill foxes and sometimes cats (but not goannas). These and other issues have been raised almost every time the published results of a study do not support the fashionable, religious belief that contemporary dingo control practices cause mesopredator releases (for examples, see^46,48–50,100,101^). As has been already discussed at length in many previous reports, however, to such criticisms we would simply respond by restating that: (1) passive tracking indices are a sensitive, robust, and valid survey technique for simultaneously monitoring relative abundances of dingoes, foxes, cats, goannas and many other terrestrial species; that (2) the arising count data do not necessarily require transformation and can be analysed in a variety of reliable ways that do not oblige researchers to use quantile regressions, occupancy models, or one particular analytical technique over another; that (3) experimental design features like large scales, treatment independence, randomisation, the presence of paired experimental controls, measurement and demonstration of a treatment effect, and stratified random sampling over multiple seasons and years, each add inferential value that cannot be matched by alternative correlative study designs that do not include these features; and (4) the reality that foxes and sometimes cats are also killed during dingo control programs has not constrained a great many other studies from claiming that dingo control causes widespread mesopredator releases despite this issue (for examples, see^24,26,71,96,102–106^). We do not elaborate on these issues here because they have already been discussed at length in many previous reports (for examples, see^12,20,56,64,67,74,86–88,107–110^). We encourage interested readers to first familiarise themselves with these reports before judging the results of our manipulative experiments to be unreliable.

## Conclusions

Australia holds the embarrassing title for the country with (by far) the most mammal extinctions in modern history, and many more threatened fauna species are predicted to become extinct over the next few decades^111,112^. There are a variety of interacting reasons for this, but one of the primary causes of mammal decline in Australia is the pervasive impacts of feral cats and European red foxes^28,113,114^. Because dingoes also threaten many mammal species and have been associated with mammal extinctions, declines, and failed reintroduction attempts in the recent past^36,115,116^, the positive indirect effects that dingoes are supposed to create by suppressing mesopredators are thought to be greater than their negative direct effects^10^. This has prompted advocacy for cessation of dingo control on grounds that doing so will supress mesopredators and stem mammal declines^14,16,91,92^. However, our large-scale and long-term manipulative experiments reveal that this view is unsupported, which has important consequences for predation management policy and practice across Australia.

Our results add to the large and growing body of long-term mensurative studies^39–41^ and experimental evidence that contemporary dingo control practices—including repeated temporary suppression of dingoes^63–66,84,85^ and also complete suppression of dingoes within fenced areas (this study)—do not produce mesopredator releases of foxes, cats or goannas. Moreover, the widespread and common absence of demonstrable negative relationships between dingoes and mesopredators at the population level (reviewed in^37,38,52^; see also^41^) further indicate that dingo control-induced mesopredator releases are unlikely to be found elsewhere. This implies that cessation of dingo control is not going to help combat the serious threat from foxes and feral cats, or that advocating for use of dingoes as some sort of biocontrol tool against cats and foxes is also misguided. We do not discount the possibility that mesopredators might be released following dingo control in some future study, or that foxes and cats may change their behaviour in the presence of dingoes without being affected numerically, but we expect such a result would be ‘the exception’ and not ‘the rule’ given the demonstrably widespread absence of evidence and evidence of absence for dingo control-induced mesopredator releases.

These results from Australia add to the growing body of evidence from other countries that mesopredator suppression or release processes are not ubiquitous. Work from Europe^117,118^, Africa^119–123^, Asia^124–126^, North America^9,127^, and South America^128,129^ also continue to report an absence of strong top-predator effects on mesopredators, with bottom-up effects appearing to be more strongly associated with mesopredator population changes. Yellowstone National Park in North America appears to be the location where these effects are most apparent, for wolves, with limited evidence for these processes available for almost all other large carnivores (^2^; see also^12^). We share the view of many^3,68,69,74,107^ that large-scale and long-term manipulative experiments are the best way of elucidating top-predators ecological roles and strongly discourage describing correlative studies as strong evidence for top-down effects. We also encourage future studies to assess prey population responses to predator manipulations as a means of bypassing the more academic predator-predator interactions and focussing on the applied issues most pertinent to conservation of threatened species.

## Methods

### Study sites

Our study was conducted at two sites near Morven (26.30S, 146.90E) and Tambo (25.20S, 146.10E) in south-central Queensland, Australia (for a map of the study sites, see^80^). Both sites are within the semi-arid Mulga Lands Bioregion which is dominated by relatively flat, undulating plains and low, timbered ranges. Mulga (*Acacia aneura*), gidgee (*Acacia cambagei*), poplar box (*Eucalyptus populnea*), coolabah (*Eucalyptus coolabah*), and silver-leaf ironbark (*Eucalyptus melanophloia*) co-dominate the taller vegetation strata at both sites. Mitchell grass (*Astrebla* spp.) is the dominate ground vegetation, interspersed with a variety of other grasses and burrs. The long-term median annual rainfall for Morven and Tambo is 510.0 mm and 518.3 mm, respectively (www.bom.gov.au), and the primary land use activities occurring at both sites are predominately sheep, goat, and some cattle grazing. At Morven, temperatures range between 46.8 °C in summer and – 9.4 °C in winter. Temperatures at Tambo range between 44.5 °C in summer and –5.6 °C in winter. Permanent natural and anthropogenic watering points exist throughout both sites. Both sites are typical and representative of the broader region of south-central Queensland where cluster fences are widespread^80^.

### Dingo exclusion fencing and lethal control

Dingo exclusion fencing was erected at each site, which consisted of a group of cooperating livestock producers that collectively erected a fence around the perimeter of their adjacent livestock properties, known locally as cluster fences (for further details, see^80,81^). The exact height and style of the fencing varied slightly from property to property, but fences were typically 1.5 m high wire mesh fences with a strained 300 mm apron and an additional one or two barbed wires on top, making the fence 1.8 m high in total. The lower 500 mm of the mesh usually consists of rectangles measuring 160 mm × 100 mm and the upper portion of the mesh consists of 160 mm squares. The fenced area at Morven is 3763 km^2^ in size with a perimeter of 424 km which was completed in January 2015. The fenced area at Tambo is 2265 km^2^ in size with a perimeter of 330 km which was completed in June 2015. These two cluster fences represent two of the largest cluster fences in the region^80^, and each enclose up to 50 individual livestock properties. Thus, their sizes are as large as or larger than all other cluster fences in western Queensland. Dingo home range and movement data collected from the sites prior to the fences being installed^130^ suggest that fenced areas of this size should contain hundreds of dingoes from dozens of packs.

Many properties on the inside of each cluster fence erected additional dingo exclusion fences of the same style around their individual property boundaries subsequent to the completion of the broader cluster fence, so that the entire area inside the cluster fences were also bisected with additional exclusion fences. Fences were erected at each site with the intention of subsequently eradicating dingoes from inside the broader cluster fenced areas and prohibiting their post-control immigration or reinvasion from the outside. These fences are also likely to provide some resistance to fox immigration, but are not expected to inhibit movement by cats or goannas in any way. Such fences do not perfectly exclude 100% of dingoes all the time (e.g. flooding or kangaroo pressure can sometimes produce temporary holes in fences which do allow some dingoes inside on occasion), but they are expected to facilitate their near-eradication, or at least enable dingoes to be removed and suppressed to functionally extinct or near-undetectable levels sufficient to raise sheep and goats with negligible predation impacts^81^. Fences at Morven were judiciously inspected and maintained on a regular basis (at least monthly) throughout the study period, with holes typically repaired within a day or two of discovery. Fences at Tambo were inspected and maintained on an irregular basis, with holes left unrepaired for several weeks following discovery in some cases.

Dingoes were controlled inside the fences by a comprehensive and intensive variety of lethal means. Repeated poison baiting with sodium fluouroacetate or ‘1080’ occurred repeatedly around April and October each year; April baiting is intended to target adult dingoes before they breed in autumn, and October baiting is intended to target pups and juveniles before they disperse in spring and summer^131^. All properties in the Morven cluster baited twice each year regardless of how many dingoes were observed or removed, except for one property which baited five times each year. Most of the properties in the Tambo cluster baited likewise, although a small number of cattle-producing properties did not participate in baiting at any time, potentially harbouring dingoes and providing a source of dispersing dingoes inside the Tambo fence.

Professional dingo trappers were employed on a repeated basis throughout the study both before and after fences were completed, along with several non-professional trappers (i.e. livestock workers). Trapping effort varied spatially and temporally inside the two fences, but was particularly intensive in the few years following the completion of the fences. Trapping effort was directed at those places and times where there was evidence of dingoes persisting. Trapping regularly occurred on all properties within the Morven cluster. Most properties in the Tambo cluster also participated in trapping, although the same cattle-producing properties that did not use poison baits likewise did not participate in trapping at any time.

Coordinated and opportunistic aerial and ground shooting activities were also undertaken at both sites, and all three forms of lethal dingo control (baiting, trapping, and shooting) were maintained at relatively high intensity throughout the entire study period. This was particularly true at Morven where there was high degree of cooperation between livestock producers within the cluster fence. However, cooperation between sheep farmers and cattle farmers at the cluster level at Tambo waned subsequent to the erection of individual fences within the cluster, producing a mosaic of smaller fenced areas where dingoes were controlled to a lesser or greater extent. Opportunistic shooting occurred inside all fenced areas at both sites.

Though experiencing short periods of high intensity control, dingoes outside the fences at both sites were generally subjected to only opportunistic shooting or sporadic trapping and baiting efforts which typically have little effect on dingo abundance over time^63^.

### Experimental design

Each site consisted of one treatment area (inside the cluster fence) where dingoes were intensively controlled and targeted for eradication, and one paired and adjacent nil-treatment area or ‘experimental control’ area of similar size (outside the cluster fence) where dingoes were only exposed to sporadic and opportunistic forms of lethal control, which is common across the broader region^63^. Experimental sites were randomly selected from the scores of other cluster fences where we could have undertaken our experiments (see^80^); and although the location of fences (or treatments) were preselected by livestock producers, allocation of treatments was essentially randomised with respect to our experimental purposes; general habitat features and historical land use etc. was relatively consistent between treatments. This experimental design was replicated at two independent sites, producing what Hone^56^ defines as a ‘classical experiment’ when results are analysed together, or an ‘unreplicated experiment’ when results are treated separately for each site. These types of experimental design yield the highest levels of inference possible for these types of studies^69,74^, but could have been improved by the inclusion of additional sites and/or additional treatments at each site. Predator population sampling was conducted in April, August and November during each year of the study beginning in November 2013 at Morven and August 2015 at Tambo. Surveys were concluded in April 2018 at Morven and November 2019 at Tambo, yielding 14 surveys at each site over 4–5 years.

### Predator density estimation (spotlighting)

Spotlighting transects were established in each treatment at each site to estimate temporal changes in predator density. Six transects inside and six transects outside the cluster fence were established at each site. Each transect was 10 km long and was located along unsealed roads or property tracks to enable consistent vehicle speeds. Spotlighting was conducted from a four-wheel-drive utility vehicle travelling at 15 km/h with a spotter standing in the back with a 100-W handheld spotlight. The spotlight was constantly moved in an approximately 160 degree arc in front of the vehicle as it moved along the road. Each predator observation recorded during the counts included details of the species, group size, and distance (m) perpendicular from the transect centre line, following typical distance sampling methods^132,133^.

We anticipated using multiple detection function models to calculate predator density estimates: a uniform key function, plus either a cosine or simple polynomial series expansion; a half-normal key function, plus either a cosine or a Hermite polynomial series expansion; and a hazard-rate key function, plus a cosine series expansion. Akaike’s Information Criterion (AIC) were to assist with selecting the most parsimonious model. Where sample sizes were too sparse (< 60 animals) to calculate specific detection functions for each transect, global detection functions were to be modelled for each site (inside vs. outside) from all transects. Density estimates were to be calculated for each species at each survey period.

### Relative abundance of predators (passive tracking indices)

Passive tracking transects (or ‘sand plots’) were established in each treatment at each site to estimate temporal changes in indices of relative abundance (PTI) of predators similar to that reported in Allen et al.^63^. This approach is endorsed by Nimmo and colleagues^51^ and has been used widely by many others to investigate this subject^25,26,65^. PTI surveys are robust to the types of temporal and spatial correlations that can affect data derived from alternative techniques because the index makes no assumptions about the number of individual animals responsible for leaving footprints on the same or adjacent sandplots, and can therefore produce valid and reliable estimates of relative abundance when applied correctly^54,86,87,134^. PTI surveys were conducted in the week preceding spotlighting efforts (described above). Sand plots were spaced on transects at 1 km intervals along unsealed roads or vehicle tracks. Care was taken to establish each transect in a mix of habitat types that were similar between treatments at each site, before randomly selecting the location of the first sand plot. Predator tracks or footprints were counted at the same time each day over three consecutive days during each survey, and sand plots were raked and smoothed clear after counting predator tracks or footprints each day. Sand plots obscured by wind, rain or other factors on a given day were removed from all analyses. Tracks were counted for each individual predator that traversed each sand plot (i.e. a continuous measure), and no attempt was made to attribute individual footprints to a specific individual predator^134^. A total of 58 and 64 sand plots were placed outside and inside the cluster fence at Morven, respectively. A total of 44 and 50 sand plots were placed outside and inside the fence at Tambo, respectively. This effort produced a total of 4538 plot nights of data for Morven and 3946 plot-nights of data for Tambo.

Predator PTI was calculated as the number of tracks per plot per night, or the mean of daily means^87,134^, which was calculated separately for each predator, treatment, site, and survey. Welch’s two-tailed T-tests were used to explore overall mean differences in predator PTI between treatments. We then assessed correlations between dingoes and foxes, cats and goannas over time, separately for each treatment and site, and used linear regression to evaluate temporal divergence of trends in predator PTI differences between treatments (i.e. inside PTI minus outside PTI) separately for each season, and all seasons combined. Accounting for season is necessary given that normal seasonal variation in predator activity is known to otherwise confound inferences and interpretations about predator PTI and relative abundance trends^86^. Severe drought at Morven in the final year of the study caused abrupt population crashes of predators and most other fauna at that time (G. Castle, unpublished data; see also Figs. 3 and 4), so we removed the surveys from this period when assessing population divergences between treatments at that site (Table 3, Fig. 5). All analyses were performed in R^135^.

### Ethics approval and consent to participate

Ethical approvals to undertake the project were provided by the Queensland Department of Agriculture and Fisheries’ Animal Ethics Committee (Approval Numbers: CA 2016/10/1010, CA 2013/10/728, and CA 2018/10/1232) and the University of Southern Queensland’s Animal Ethics Committee (Approval Number: 16REA016). All procedures described in this report were performed in accordance with these approvals. The study complies with all relevant institutional and national guidelines.

### ARRIVE guidelines

We confirm that our experiment is reported in accordance with ARRIVE guidelines (https://arriveguidelines.org).

## Data Availability

The datasets supporting the conclusions of this article are included within the article.
